# Network Analysis Identifies Proinflammatory Plasma Cell Polarization for Secretion of ISG15 in Human Autoimmunity

**DOI:** 10.4049/jimmunol.1600624

**Published:** 2016-06-29

**Authors:** Matthew A. Care, Sophie J. Stephenson, Nicholas A. Barnes, Im Fan, Alexandre Zougman, Yasser M. El-Sherbiny, Edward M. Vital, David R. Westhead, Reuben M. Tooze, Gina M. Doody

**Affiliations:** * Section of Experimental Haematology, Leeds Institute of Cancer and Pathology, University of Leeds, Leeds LS9 7TF, United Kingdom;; † Bioinformatics Group, Institute of Molecular and Cellular Biology, University of Leeds, Leeds LS2 9JT, United Kingdom;; ‡ Haematological Malignancy Diagnostic Service, Leeds Teaching Hospitals National Health Service Trust, Leeds LS9 7TF, United Kingdom;; § Section of Oncology and Clinical Research, Leeds Institute of Cancer and Pathology, University of Leeds, Leeds LS9 7TF, United Kingdom;; ¶ Leeds Institute of Rheumatic and Musculoskeletal Medicine, University of Leeds, Leeds LS2 9JT, United Kingdom; and; ‖ National Institute for Health Research Leeds Musculoskeletal Biomedical Research Unit, Leeds Teaching Hospitals National Health Service Trust, Leeds LS9 7TF, United Kingdom

## Abstract

Plasma cells (PCs) as effectors of humoral immunity produce Igs to match pathogenic insult. Emerging data suggest more diverse roles exist for PCs as regulators of immune and inflammatory responses via secretion of factors other than Igs. The extent to which such responses are preprogrammed in B-lineage cells or can be induced in PCs by the microenvironment is unknown. In this study, we dissect the impact of IFNs on the regulatory networks of human PCs. We show that core PC programs are unaffected, whereas PCs respond to IFNs with distinctive transcriptional responses. The IFN-stimulated gene 15 (ISG15) system emerges as a major transcriptional output induced in a sustained fashion by IFN-α in PCs and linked both to intracellular conjugation and ISG15 secretion. This leads to the identification of ISG15-secreting plasmablasts/PCs in patients with active systemic lupus erythematosus. Thus, ISG15-secreting PCs represent a distinct proinflammatory PC subset providing an Ig-independent mechanism of PC action in human autoimmunity.

## Introduction

The polarization of immune cell populations for the secretion of distinct profiles of extracellular signaling molecules to drive and modulate immune responses has emerged as a recurrent feature across adaptive and innate immunity. For the B cell lineage, initial descriptions centered on an immune regulatory function and the secretion of IL-10 (1, 2); more recently, additional secretory capacity has been identified in murine models, including the secretion of TNF-α, NO, IL-17, and IL-35 (3–5).

An important feature that distinguishes the B cell lineage from other cellular populations is the capacity for fundamental reprogramming toward dedicated high-level secretory capacity associated with the plasma cell (PC) state. This high-level secretory activity is first acquired in proliferating PC precursors, known as plasmablasts, and because exit from cell cycle is the principal feature separating these cell states, both plasmablast and PC populations are referred to as Ab-secreting cells (ASCs). The canonical secretory product of ASCs is their specific Ig; however, the importance of alternate bioactive secretory products is increasingly being appreciated (6). Such non-Ig secretory activity in ASCs has been primarily characterized in murine model systems highlighting specific relationships between selected pathogens and ASC-derived cytokine secretion. Although in some instances non-Ig secretory functions have been shown to develop during differentiation to the PC state (3–5, 7), in other instances, the rapidity with which cytokine-secreting PCs emerged after infectious challenge suggested the possibility that the response may have developed in previously established ASCs (8). An ability of ASCs to respond to environmental cues by acquiring polarized immune modulatory functions would resemble responses in macrophage/monocytes, in which functional polarization is determined by the prevailing milieu and can represent a labile phenotype within a cell population (9).

In humans, the importance of non-Ig secretory activity from ASCs is less well characterized. PCs are, however, maintained in a variety of distinct microenvironmental states including both primary and secondary lymphoid tissue and tissues undergoing acute and chronic inflammation. The latter environments are frequently accompanied by polarization of other lymphoid and innate immune cell populations for specific secretory activity.

One of the most important autoimmune conditions in which ASCs play a role is systemic lupus erythematosus (SLE). SLE shows a strong pathogenic association with both autoantibodies and IFN responses (10). Evidence for IFNs as immediate drivers of such pathology is provided by the monogenic IFNopathies, in which sequence variants in diverse upstream regulators lead to exaggerated IFN responses and convergence on autoimmune features closely related to SLE (7). Type 1 IFN in particular secreted by plasmacytoid dendritic cells has been identified as a factor that can enhance the generation of PCs in vivo (11), and we and others (12–14) have shown that IFN-α can contribute to the generation and maintenance of long-lived human PCs in vitro. Although autoantibodies play an important role in SLE and other autoimmune pathology, there is considerable interest in defining potential mechanisms linking the B cell lineage, including ASCs, to autoimmune pathology that are independent of Igs (15).

An important question also relates to the interaction between type 1 and type 2 IFN-mediated immune responses. Again, monogenic diseases have been informative in this regard, and the type 1 IFN-responsive gene IFN-stimulated gene 15 (*ISG15*) has emerged as a potentially critical bridge. ISG15 has a complex biology linked, on the one hand, to covalent intracellular modification of target proteins, ISGylation, a process analogous to ubiquitinylation, and on the other hand to a role as an extracellular signaling molecule/cytokine (16). In the latter role, ISG15 has been known for several years as an activator of NK cells and a driver of IFN-γ secretion (17, 18). In the context of monogenic disease affecting ISG15 in the patients so far described, the primary determinant of disease phenotype was the presence or absence of *Mycobacterium* bacillus Calmette-Guérin (BCG) vaccination (19). In the presence of BCG vaccinations, patients presented with disseminated mycobacterial infection attributable to the failure of ISG15-dependent extracellular signaling and IFN-γ–driven immune responses. In contrast, the absence of BCG vaccination in a second set of patients revealed a critical function for intracellular ISG15 in the inhibition of type 1 IFN responses and mutations in *ISG15* led to an IFNopathy (20).

Given the importance of ASCs in SLE, and our identification of IFN-α as a factor capable of sustaining long-lived PC survival, we were motivated to dissect the effect of IFN-α in more detail. In this study, using detailed gene network analysis of the plasmablast to PC transition, we show that IFN-α provides a potent environmental signal driving a distinct program of gene expression in PCs, mirroring patterns observed in SLE, without globally impacting on the fundamental processes of entry into cell cycle, quiescence, or the completion of secretory reprogramming. We identify the sustained and functional induction of *ISG15* and components of ISGylation machinery (16) as a primary effect of IFN-α on human ASCs. Critically, this is accompanied by direct secretion of ISG15, a feature that is also observed in primary human ASCs cultured in the presence of IFN-α and spontaneously from plasmablasts of patients with SLE. These data characterize a new population of human inflammatory PCs polarized for the secretion of the proinflammatory mediator ISG15, identifying an Ig-independent mechanism of PC action in human autoimmunity. This establishes the basic principal that human PCs are subject to polarization by the microenvironmental milieu to acquire the capacity for immune modulation.

## Materials and Methods

### Abs and reagents

Reagents were human IL-2 (Roche); IFN-γ (Sigma-Aldrich); IL-6, IFN-α, and IL-21 (PeproTech); goat anti-human F(ab′)_2_ fragments (anti-IgM and -IgG; Jackson ImmunoResearch Laboratories); HybridoMax hybridoma growth supplement (Gentaur); Lipid Mixture 1, chemically defined (200×) and MEM Amino Acids Solution (50×; Sigma-Aldrich); CountBright beads (Invitrogen); and 7-aminoactinomycin D (BD Biosciences). The Abs used for flow cytometry were Viogreen-conjugated anti-CD3 (BW264/56; Miltenyi Biotec), PE-conjugated anti-CD19 (LT19; Miltenyi Biotec), V450-conjugated anti-CD20 (2H7; eBioscience), A700/FITC-conjugated anti-CD27 (M-T271; BD Biosciences), PE-Cy7–conjugated anti-CD38 (HB7; BD Biosciences), and allophycocyanin-conjugated anti-CD138 (B-B4; Miltenyi Biotec). Abs used for biochemical/proteomic evaluation were anti-ISG15 (Proteintech and [3E5] Santa Cruz Biotechnology), anti-SYK (D1I5Q; Cell Signaling Technology), histone H3 (Abcam), peroxidase-conjugated goat anti-rabbit IgG (H+L) (Jackson ImmunoResearch Laboratories), and anti–β-actin (AC15; Sigma-Aldrich). Immunohistochemical staining was performed using mouse monoclonal anti-ISG15 (F9; Santa Cruz Biotechnology). The ELISPOT assay employed a mouse anti-ISG15 (3E5; Santa Cruz Biotechnology) capture Ab followed by a primary rabbit anti-ISG15 (ProteinTech) and secondary peroxidase-conjugated goat anti-rabbit detection Abs (Jackson ImmunoResearch Laboratories) and TMB substrate (Mabtech).

### Cell isolation and culture

Peripheral blood was obtained from healthy donors or patients with a confirmed diagnosis of SLE after informed consent. Approval for this study was provided by the U.K. National Research Ethics Service via the Leeds East Research Ethics Committee (approval reference: 07/Q1206/47 as well as 10/H1306/88 for collection of samples and data from patients with SLE).

Mononuclear cells were isolated by Lymphoprep (Alere) density gradient centrifugation. Total B cells were isolated from healthy donor samples by negative selection with the memory B cell isolation kit (Miltenyi Biotec). Plasmablasts were identified by surface staining for CD19^+^CD38^+^CD27^+^ and sorted using a BD Biosciences Influx. Culture conditions for the generation of plasmablasts and PCs from peripheral blood human B cells have been described in detail elsewhere (12). At day 6, cells were treated with or without IFNs as indicated.

Day 6 plasmablasts were reseeded at 1 × 10^6^ in transwells in media supplemented with IL-6 (10 ng/ml), IL-21 (50 ng/ml), HybridoMax hybridoma growth supplement (11 μl/ml), Lipid Mixture 1, chemically defined (5 μl/ml), and MEM amino acid solution (20 μl/ml) on γ-irradiated M2-10B4 cells (4 × 10^4^/well) with either IFN-α (200 IU/ml) or IFN-γ (200 IU/ml).

### Flow cytometry

Cells were stained with directly conjugated Abs and analyzed on an LSR II flow cytometer (BD Biosciences). Live cells were gated using forward light scatter/side scatter characteristics and exclusion of 7-aminoactinomycin D (BD Biosciences). Controls included isotype-matched conjugated Abs. Absolute cell counts were performed with CountBright beads (Invitrogen). Data analysis was performed using FACSDiva Software 8.0 (BD Biosciences).

### Gene expression data acquisition and analysis

Total RNA was extracted using TRIzol (Invitrogen), subjected to DNAseI treatment (DNA Free; Ambion), and subsequently amplified using the Illumina TotalPrepTM-96 RNA Amplification Kit (Life Technologies). Labeled cRNAs were hybridized onto HumanHT-12 v4 Expression BeadChips (Illumina) according to the manufacturer’s instructions and scanned with the Illumina BeadScanner. Illumina GenomeStudio Gene Expression Module was employed for initial data processing, and probes were annotated with approved symbols (HUGO; version 2015/01/14) and further processed with Lumi (21, 22). Probes not detected on three or more arrays were removed and the remaining data quantile normalized (*n* = 21,209 probes). A linear model was fitted to the gene expression data using the R Limma package (23). Differentially expressed genes between the contrasts were gauged using the Limma empirical Bayes statistics module, adjusting for multiple testing using Benjamini and Hochberg correction.

### Network construction and module analysis

The top 2000 most variant probes were used to construct a coexpression network using the weighted gene coexpression network analysis (WGCNA) approach (24). An adjacency matrix was constructed using absolute Pearson correlation coefficient ^ 8 (soft threshold) and converted to a topological overlap matrix (TOM). Modules were identified using cutreeDynamic with deepSplit = True, minClusterSize = 8, cutHeight = 0.995, and method = Tree. Similar modules were merged using mergeCloseModules with a cutHeight = 0.2. Generated modules between conditions were compared using a Fisher exact test to give a heat map showing the number of overlapping genes between two treatment types, with the overlaps shown as numbers and the significances as the color scale (−log_10_ *p* values). These overlap heat maps were used to recolor the WGCNA modules so that maximally overlapping modules had the same color.

### Network visualization

Node and edge lists output by WGCNA were converted (retaining 50,000 most significant edges) and imported into the Gephi package (25). Nodes were colored according to the WGCNA module colors, node size scaled according to Betweenness-centrality, edges colored by WGCNA TOM weight, and graph layout was carried out using Gephi's ForceAtlas 2 (LinLog mode, Prevent-Overlap, Edge-Weight-Influence = 1, Scaling = 0.05, Gravity = 1, Tolerance = 5000, Approximation = 1.2).

### Signature enrichment analysis

A data set of 14,154 gene signatures was created by merging signatures downloaded from http://lymphochip.nih.gov/signaturedb/ (SignatureDB), http://www.broadinstitute.org/gsea/msigdb/index.jsp MSigDB V5 (MSigDB C1–C7 and H; excluding C5. With MIPS signatures from version 3.1 and PID signatures from version 4 added back), http://compbio.dfci.harvard.edu/genesigdb/ Gene Signature Database V4 (GeneSigDB), UniProt keywords (downloaded using biomart), and seven papers (26–35).

A gene ontology gene set was created using an in-house python script. This parses a gene association file (http://geneontology.org/page/download-annotations) to link genes with ontology terms and then uses the ontology structure (.obo file; http://purl.obolibrary.org/obo/go.obo) to propagate these terms up to the root. The resultant gene set contained 19,599 terms.

The gene-ontology and gene-signatures sets were merged to give a final signature set of 33,753 terms. Enrichment of gene lists for signatures was assessed using a hypergeometric test, in which the draw is the gene list genes, the successes are the signature genes, and the population is the genes present on the platform.

### Module signature enrichment comparison

The expression of the 2000 probes used for network construction was analyzed across the treatment time courses, summing the log_2_ expression values between each consecutive time point and allowing the overall trend (up/downregulated) of each gene to be assessed. The WGCNA module/connectivity files were processed to output gene lists, retaining probes with an SD (across time course) >0.2. Probes in each module were split into those that had an up/down expression trend, giving two module gene lists per WGCNA module; lists with less than five genes were discarded.

The genes present in each module gene list were used for signature enrichment analysis. A representative selection of the most enriched signatures was compared across the modules and treatment types. The enrichment z-scores were retrieved for all modules per representative signature, z-scores were set to 0 when the *p* value >0.05, and any module with no enriched signature was removed. The matrix was then hierarchically clustered (Pearson, Complete) using GENE-E (https://www.broadinstitute.org/cancer/software/GENE-E/).

### Connectivity versus expression

Intramodular connectivity and gene expression were compared between pairs of treatments. The WGCNA modular connectivity was scaled (0–1) within each module. The Limma T-Statistics was used as a measure of differential gene expression between treatments at each time point.

### Western blotting and proteomic analysis

At the indicated time point, primary cells were harvested, washed with PBS, and lysed in reducing Laemmli buffer to generate whole-cell lysates.

Alternatively, plasmablasts that had been cultured in the presence of IFN-α for 24 h were lysed in either RIPA or Nonidet P-40 buffer containing protease inhibitors. Immmunoprecipitates were prepared from these lysates using Abs against ISG15, SYK, or isotype controls. Samples were separated SDS-PAGE and then transferred to nitrocellulose. Abs used to detect ISG15, SYK, and β-actin were subsequently incubated with the immunoblot and detected by ECL (SuperSignal West Pico Chemiluminescent Substrate; Thermo Scientific). ISG15 immunoprecipitates prepared from IFN-α–stimulated plasmablasts were subject to trypsin digestion by the Strap method, consequent liquid chromatography tandem mass spectrometry, and data processing performed as previously described (36).

### ELISPOT

For detection of ISG15 secretion, polyvinylidene difluoride plates were activated with 70% ethanol, washed with sterile water, and then coated with mouse anti-human ISG15 (1.5 μg/well in sterile PBS) capture Ab overnight at 4°C. The plates were then washed with sterile PBS and blocked with IMDM containing 10% FBS. Plasmablasts were added at the indicated cell number in media containing IMDM, 10% FBS, IL-21 (50 ng/ml), and IL-6 (10 ng/ml) in the presence or absence of IFN-α (100 U/ml). Plates were then incubated for 20–24 h in a humidified incubator. Cells were removed from the plate and followed by a PBS wash. To detect ISG15 secretion, rabbit anti-human ISG15 was added to each well (0.1 μg/well in PBS containing 0.5% BSA) and the plates were incubated for 2 h at room temperature. Alternatively, mouse IgG was used to coat the plates as a control for nonspecific spot formation. The plate was then washed and peroxidase-conjugated goat anti-rabbit Ab added (diluted 1:4000 in PBS containing 0.5% BSA) for 1 h at room temperature. The plate was then washed, and spots were detected by addition of TMB substrate. Images were captured using an AID EliSpot Reader System with software version 5.0 (Autoimmun Diagnostika). For detection of human IgG secretion, the Human ELISpot IgG Plus kit (Mabtech) was used. The assay was performed as described in the manufacturer’s protocol.

### Data access

The complete set of array data has been submitted to the National Center for Biotechnology Information Gene Expression Omnibus (http://www.ncbi.nlm.nih.gov/geo) under accession number GSE75007.

## Results

### Impact of inflammatory cytokines on PC generation

IFNs are central to both effective immune responses and pathogenic autoimmunity and represent likely candidates as mediators of the functional polarization of PC populations. Type 1 IFN has been shown to support PC differentiation (11, 13), and we previously determined that inclusion of IFN-α at the plasmablast stage contributed to the emergence of long-lived human PCs in vitro (12). Developing plasmablasts also have the potential to be exposed to IFN-γ, for example, in the context of production by T follicular helper cells in the germinal center (37). Furthermore, it remained unknown whether IFN exposure impacted on the core processes of PC differentiation or acted to impose an additional distinct functional signature on a conserved differentiation pathway. In SLE, it is well established that the peripheral blood can show signatures indicative of an active IFN response (30, 38–41), and such responses may in part derive from circulating ASC populations at the plasmablast to PC transition. We therefore focused on a detailed analysis of the effects of IFNs at this final differentiation step of the B cell lineage.

**FIGURE 1.**
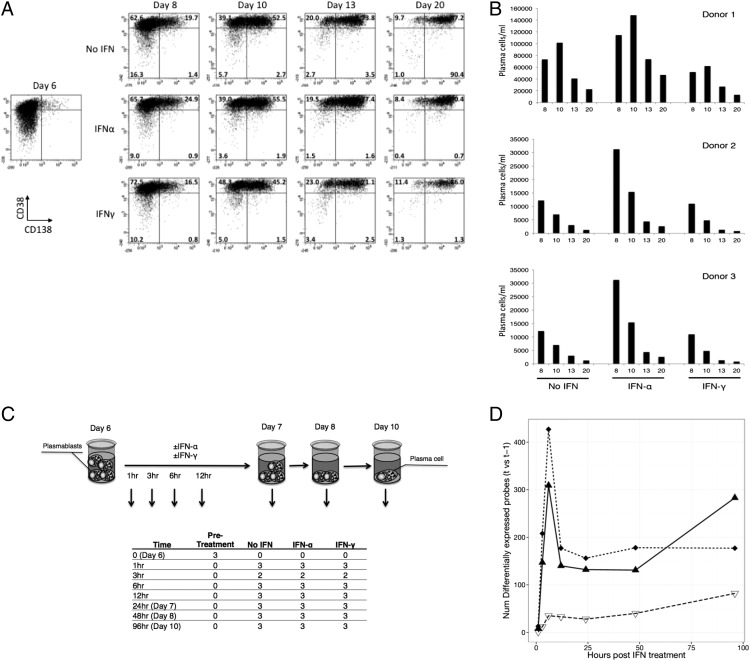
Characterization of the impact of IFN on in vitro PC generation. (**A**) IFN exerts minimal influence on the phenotypic maturation of human PCs as assessed by the level of CD38 and CD138 acquisition over a period of 3 wk. Representative FACS plots gated on viable cells from a single donor are shown. (**B**) Enumeration of absolute numbers of viable cells obtained at the indicated time in culture with standard media alone or with added IFN-α or IFN-γ. Data from three donors are displayed. (**C**) Schematic of time-course evaluation of gene expression during the transition from plasmablast to PC. Data are derived from samples generated from three donors at eight time points. (**D**) The number of differentially expressed genes (false discovery rate 0.05) for the contrasts IFN-α versus no IFN (dotted line/diamonds), IFN-α versus IFN-γ (solid line/triangles), and IFN-γ versus no IFN (dashed line/open triangles) at each time point.

To address this question, we employed our differentiation model focusing on ASCs undergoing the transition from an actively cycling population (plasmablasts) to cell cycle quiescence (PCs), as previously defined in our system (12), and undertook a detailed time-course analysis of gene expression during this transition. Phenotypic maturation was monitored by the loss of B cell marker CD20 and concomitant acquisition of PC markers CD38 and CD138. Within 48 h of initiating the plasmablast to PC transition in media, containing IL-6 and IL-21 alone or supplemented with either IFN-α or IFN-γ, a CD38^hi^CD138^+^ population was readily detectable in all culture conditions (Fig. 1A). The proportion of cells with PC phenotype increased with time, reaching ∼90% by day 20. Overall, there were no consistent differences observed in the cell surface phenotypes obtained in the three conditions. A reproducible feature of our previous experiments was the ability of IFN-α to enhance the number of viable PCs (12). In contrast to IFN-α, IFN-γ failed to increase the number of PCs, even in comparison with cells maintained only with added IL-6 and IL-21 (Fig. 1B).

For gene expression analysis during the transition from plasmablast to PC, RNA was sampled at five time points during the initial 24 h after exposure to IFN followed by subsequent sampling at 48 and 96 h (Fig. 1C). To gain an overview of the impact of IFN, we assessed the overall pattern of the variation in gene expression. Notably, IFN-α stimulation produced a profound change at the early time points, showing a maximal change of 427 genes (versus no IFN; false discovery rate 0.05) at 6 h and reaching a plateau from 12 h onwards (Fig. 1D, Supplemental Table I). Incubation with IFN-γ produced an early change in gene expression that was sustained and gradually increased over the 5-d period. Both IFN-α and IFN-γ showed nearly equivalent induction of shared targets genes such as *STAT1* (6.3 versus 4.6 median fold induction, respectively).

**FIGURE 2.**
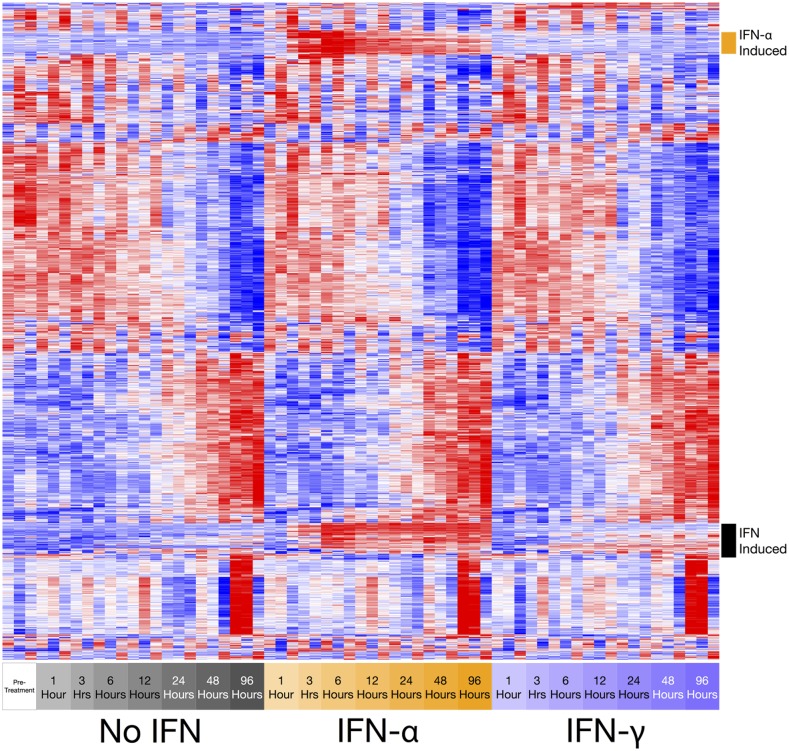
Longitudinal gene expression profiling of differentiating ASCs exposed to IFN. Hierarchically clustered heat map representation of global gene expression profiles derived from timed intervals following exposure of human plasmablasts to no IFN, IFN-α, or IFN-γ. Changes in gene expression attributable to IFN stimulation are indicated. Row-wise, Pearson correlation, complete linkage.

These patterns of overall differential gene expression indicate that IFN stimulation has the potential to drive changes in the regulatory networks operating in plasmablasts, which might alter the reprogramming pathways that define PC identity. To further profile the global transcriptional changes, we performed hierarchical clustering analysis on the 2000 most variant probes (Fig. 2). The resulting patterns demonstrated that the majority of these gene probes displayed similar kinetics, regardless of culture conditions. There were large cohorts of genes, including prototypical B cell and PC identifiers, components of secretory pathways, and genes associated with metabolic function for which the overall pattern of regulation was preserved in the presence or absence of IFN. Among these genes, there were subtle differences in the kinetics for individual donors, but the overall picture remained consistent. However, as anticipated from the initial analysis, there were blocks of genes that showed specific changes in response to either IFN-α or IFN-γ.

### Application of WGCNA to define coregulated gene modules at the plasmablast to PC transition

The basic underlying gene regulatory networks and master regulators associated with the reprogramming that accompanies differentiation to the PC state have been extensively documented (42). However, recent studies employing systems-scale network analysis in other immune cell lineages have identified novel hub genes responsible for coordinating molecular circuits that operate in context-specific settings (43). To provide additional insight into the genes and pathways that feature in differentiating ASCs at the plasmablast to PC transition, we performed gene coexpression network analysis on our temporal transcriptome data.

**FIGURE 3.**
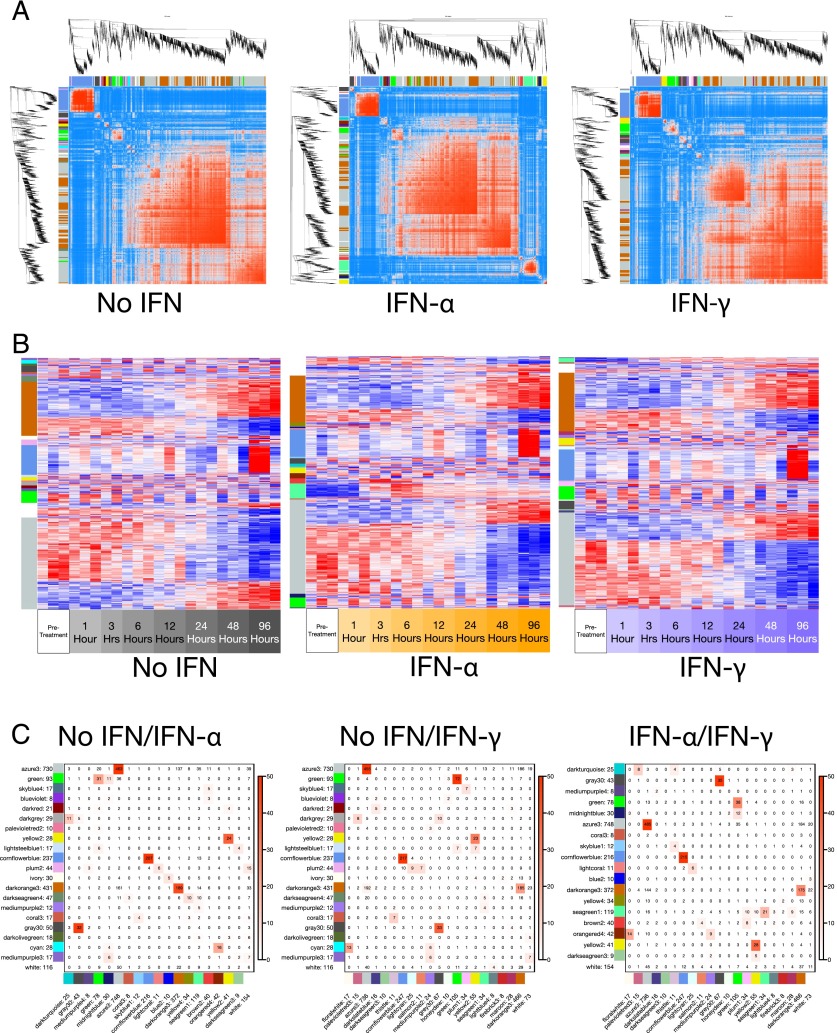
Construction of gene coexpression networks and module membership. (**A**) Identification of coexpressed transcripts by pairwise correlation. Highly connected genes are displayed in a hierarchically clustered TOM. (**B**) Gene expression patterns within each WGCNA module. Module colors were assigned so that maximally overlapping modules had the same color; see (**C**). (C) Heat map showing the number of overlapping genes between modules within two treatment types, with the overlaps shown as numbers and the significances as the color scale (−log10 *p* values; Fisher exact test). Comparisons between media-treated versus IFN-α–treated samples, media-treated versus IFN-γ–treated samples, and IFN-α–treated versus IFN-γ–treated samples.

Using the WGCNA TOM, we evaluated the relationship among all gene pairs to establish sets of interdependent genes. These sets or modules will contain common and signaling-specific molecular circuits, allowing a greater resolution of the networks controlling PC biology. Modules were determined based on hierarchical clustering of genes with coordinated expression and assigned individual colors to denote membership (Fig. 3A, Supplemental Table II). The expression of closely connected genes belonging to network modules was then plotted as heat maps to follow the temporal patterns of expression change (Fig. 3B). The largest modules indicated by “dark orange” and “azure” showed similar kinetics across the three conditions and varied primarily in the magnitude of change. As module membership is determined by expression similarity in an unsigned fashion (absolute correlations), these modules contain genes that are both coordinately upregulated and downregulated during differentiation. In contrast, genes that are acquired in a relatively abrupt manner at a late time point populate the third largest module “cornflower blue.” The remaining smaller modules are more variable and may in part reflect donor differences.

To determine the extent of overlap between modules in the three differentiation conditions, we performed Fisher exact tests (Fig. 3C). The results are illustrated as a heat map showing the significance of overlap between module memberships in the pairwise comparison of each of the three culture conditions. A high degree of similarity between conditions was detected for modules such as “azure,” “cornflower blue,” “dark orange,” and “gray.” Smaller modules also exhibited significant similarity. Notably, IFN-associated modules “brown” and “sea green” were readily identified by the lack of overlap with modules derived from cells cultured without IFNs. These modules showed partial overlap in the comparison between IFN-α and IFN-γ.

### Signature enrichment analysis to establish module-associated biology

**FIGURE 4.**
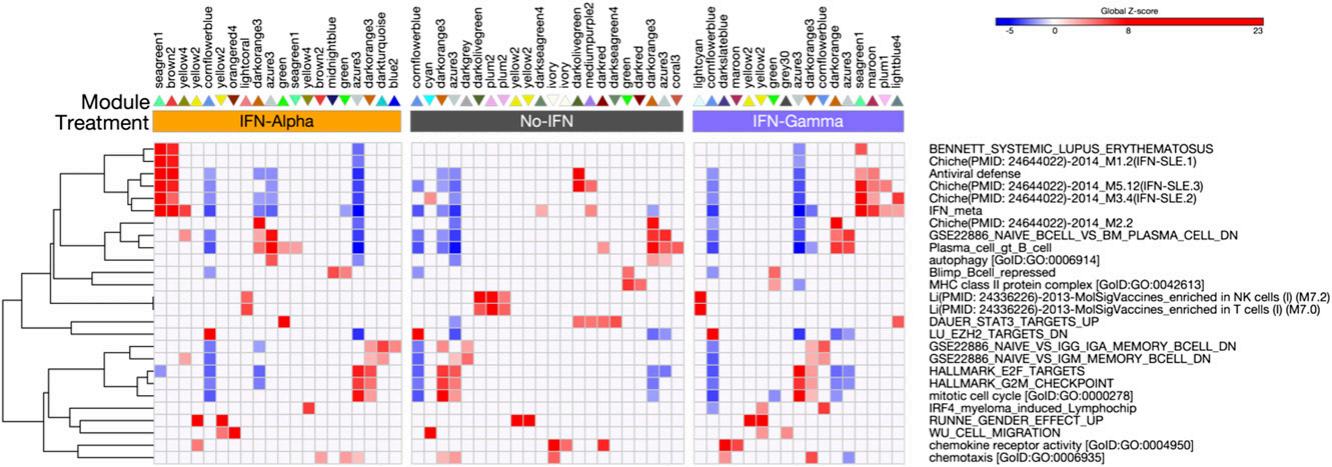
Functional annotation of modules detected in differentiating ASCs. Modules were resolved into groups showing up/downregulation of genes (retaining those with SD >0.2 across time course). These were assessed for enrichment of gene signatures (*p* < 0.05). Heat map shows hierarchical clustering (Pearson/complete) of a representative selection of enriched signatures, displaying enrichment/depletions as significant z-scores (*p* > 0.05 set to z-score of 0). Displayed modules were restricted to ones with membership of at least five genes, and only modules with at least one signature enriched were retained. Modules are listed by name with directionality of gene expression change indicated by the arrowhead of the appropriate color. Enriched GO/signature terms are specified on the right of the heat map, and z-scores of enrichment or depletions are indicated with a blue-red color scale.

To assess the underlying biological processes operating within each module, we performed signature enrichment analysis. In this instance, we separately evaluated induced and repressed genes within modules and restricted depiction of modules to those that contained at least five genes within a given gene ontology (GO) category (Fig. 4). Consistent with a defining feature of PC maturation, GO terms related to cell cycle were prominently associated with the downregulated component of “azure” and “dark orange” modules. Conversely, the upregulated genes in these modules were associated with a defined PC signature and included numerous components of the unfolded protein response, such as *XBP1*, *HERPUD1*, and *DNAJB9*. In addition to these shared modules linked to cell cycle exit and secretory adaptation, B cell genes repressed by BLIMP1 were associated with shared module “green.” This confirmed that the core PC processes of entry into cell cycle quiescence and adaption for high-level secretory state were conserved in the presence or absence of IFN stimulation. Genes associated with autophagy were also commonly upregulated across all three conditions and linked to the “azure” and “dark orange” modules, which agrees with the reported requirement for this process in long-term PC survival (44). Another shared feature across all conditions was the association of “cornflower blue” (up) with EZH2 targets, consistent with data describing a role for this epigenetic modifier in maintaining a germinal center phenotype and repressing transition to the PC state (45–47). Importantly, this analysis also showed that the IFN-related “brown” and “sea green” modules, particularly in the context of IFN-α, showed highly significant overlap with modules identified by Chiche et al. (30) as predictive signatures of disease activity in peripheral blood samples from patients with SLE.

### Construction of regulatory networks defining core and inflammatory circuits

**FIGURE 5.**
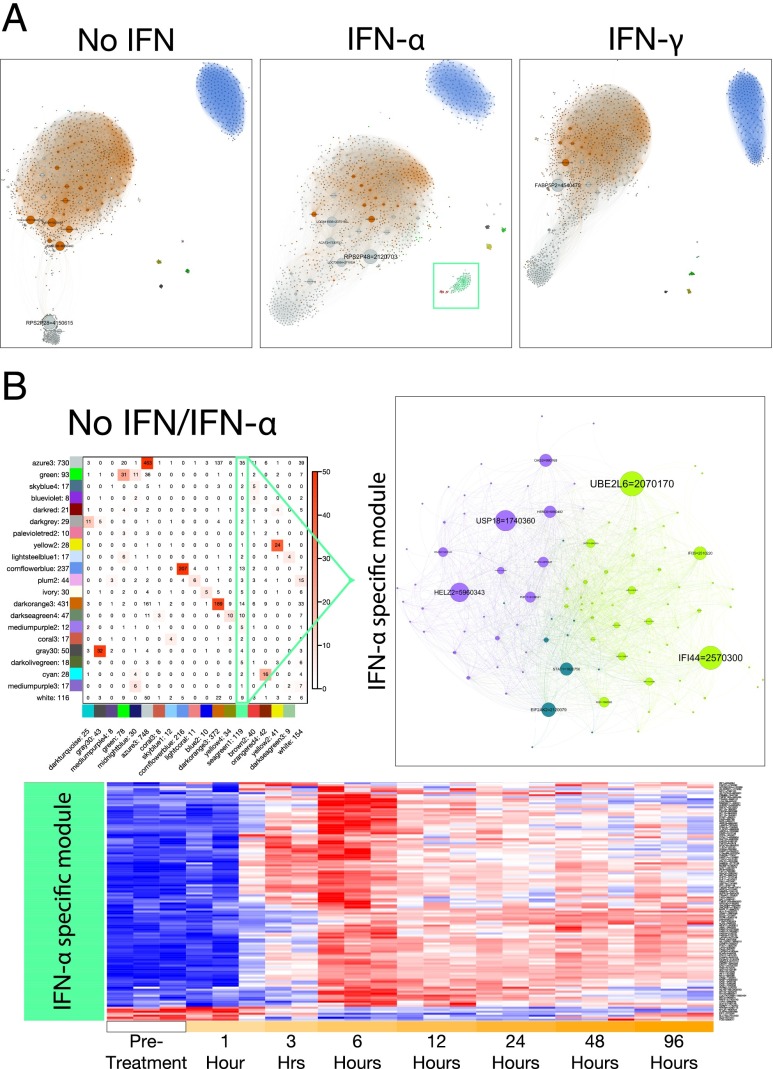
Network analysis of differentiating ASCs reveals imposed IFN program. (**A**) Networks constructed from WGCNA analysis of the 2000 most variant probes. The 50,000 most significant edges (TOM) were visualized using Gephi. Node color, WGCNA module color; node size, betweeness-centrality; and edge color, TOM value. Green box surrounds the sea green 1/brown 2 IFN-α modules. (**B**) IFN-α drives a distinct module of gene expression in differentiating ASCs (heat map from Fig. 3C). The IFN-α–specific network: node/edge color, Gephi modularity class; node size, betweeness-centrality. The heat map shows the expression of the IFN-α–specific module probes.

We next used the 50,000 most significant topological overlap values (edges) to construct a gene network, identifying hub genes within modules associated with the genetic programs of PCs generated under the three different conditions (Fig. 5A). Although the overall structure of large module networks was generally intact in the three conditions, there were distinctive features, most readily observed in the visualization of the “gray” module. This was accompanied by a change in the relative connectivity of hub genes, with prominent nodes of *RPS2P28*, *RPS2P48*, and *FABP5P2*, respectively. Interestingly, these genes, and many of the other highly connected hubs within this module, are pseudogenes, which have received increasing attention for potential roles in gene regulation and disease (48). Also conspicuous were several small, isolated networks, including the “sea green” module associated with IFN-α exposure (surrounded by green box).

We next investigated the IFN-specific “sea green” network in more detail (Fig. 5B). Many of the hub genes represent archetypal IFN-responsive genes that tended to reach peak expression 6 h poststimulation. There was also a small subset of genes, including *EIF1*, *SLC1A5*, *GLS*, and *FOS*, that were transiently repressed in the presence of IFN-α. The fact that two of these are related to glutaminolysis suggests that IFN may be able to impinge on the metabolic control of PC differentiation. Moreover, the analysis points to the transcriptional cofactor *HELZ2* as a central hub in the IFN-α response, providing an unexpected link to a regulator of adipocyte differentiation and hepatic lipogenic gene expression (49, 50).

**FIGURE 6.**
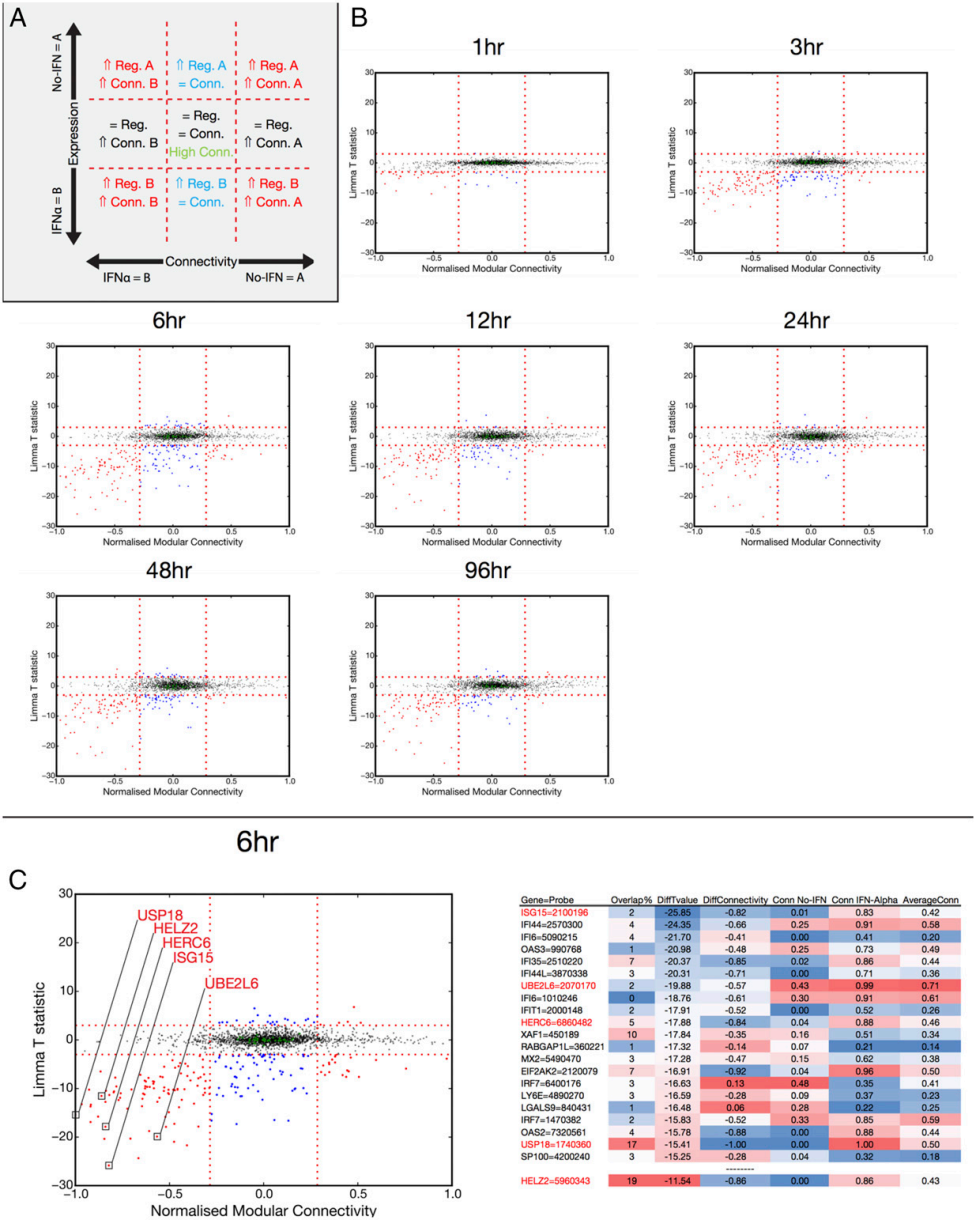
Connectivity measure of differentially expressed genes identifies the ISG15-related pathway as a key feature of ASCs generated in the presence of IFN-α. (**A**) Diagrammatic representation of plots generated to assess intramodule gene connectivity versus gene expression. (**B**) Observed changes in connectivity and expression patterns following IFN-α treatment at the indicated time points. *x*-axis, normalized (0–1) intramodular connectivity; *y*-axis, Limma T-statistic for IFN-α versus no IFN. Vertical dotted lines at ±0.285 connectivity; horizontal dotted lines at ±3 T-statistic. Blue dots, significant expression change; red dots, significant expression/connectivity change; green dots, highly connected. (**C**) Detailed view of connectivity/expression changes induced by IFN-α treatment at 6 h. The genes with the greatest change in gene expression value are shown in the table on the right, a selection of which are annotated on the plot on the left.

Large changes in the magnitude of transcript level are likely to signify importance during a response, but may not be the only indication of being a crucial contributor. A member of a particular network or module may change its correlation partners over time, allowing a diversity of outcomes by combinatorial usage. To explore the relationships between genes within modules during the PC differentiation process in the context of IFN-α, we analyzed the gene connectivity patterns versus expression level at each of the sampled time points (Fig. 6). In these plots, genes positioned centrally show neither change in expression nor change in degree of module connectivity; however, these can still be highly connected genes that may function during this phase of PC maturation irrespective of additional environmental stimuli. An example is the transcription factor *KLHL6*, which has previously been implicated in B cell receptor signaling and is recurrently mutated in chronic lymphocytic leukemia (51, 52). Genes that do not appreciably change expression between conditions, but change degree of connectivity may exhibit different roles as the plasmablasts continue to differentiate under the influence of inflammatory cytokines. *IL10RA* falls into this category; IL-10 is known to potentiate PC differentiation (53–55), but our data suggest that its effect may be altered in the presence of IFN-α. Additionally there is a cohort of genes that change in expression, but retain a similar degree of module connectivity. Importantly, although the degree of module connectivity may not change, the individual genes contributing to connectivity often change significantly. Thus, a gene may show no overall change in degree of connectivity but entirely change the genes with which it is connected (Supplemental Table II). As anticipated, both *STAT2* and IFN regulatory factor (*IRF*) *7* are examples of this category and are highly induced during exposure to IFN-α. Although maintaining a similar degree of module connectivity *STAT2* and *IRF7* fundamentally alters the nature of genes to which they connect, sharing only 2 and 4% of the top 100 most connected genes between ASCs differentiating in the presence or absence of IFN-α. These transcription factors are central to IFN-mediated signaling and, as expected, switch their connectivity to IFN-regulated genes upon exposure to IFN-α.

In addition to genes such as *STAT2* and *IRF7* that switch connected genes without altering their degree of connectivity, a substantial number of genes show changes in both expression and connectivity patterns in a sustained fashion over time. We have highlighted examples from the peak time point (*t* = 6 h) of differential gene expression in ASCs treated with IFN-α. Included in this group is the hub gene *HELZ2*, giving further support to context-specific function for this transcriptional regulator. Interestingly, the gene exhibiting the greatest difference in both expression level and connectivity in IFN-α–treated plasmablasts was *ISG15*. This was accompanied, in this group of IFN-α–induced genes, by the several enzymatic components required for coupling of ISG15 to intracellular targets. The large shift in connectivity in this group implies that ISG15-related function may play a particular role in plasmablasts responding to an inflammatory environment.

### The ISG15-conjugating system is a dominant functional attribute of IFN-driven ASCs

**FIGURE 7.**
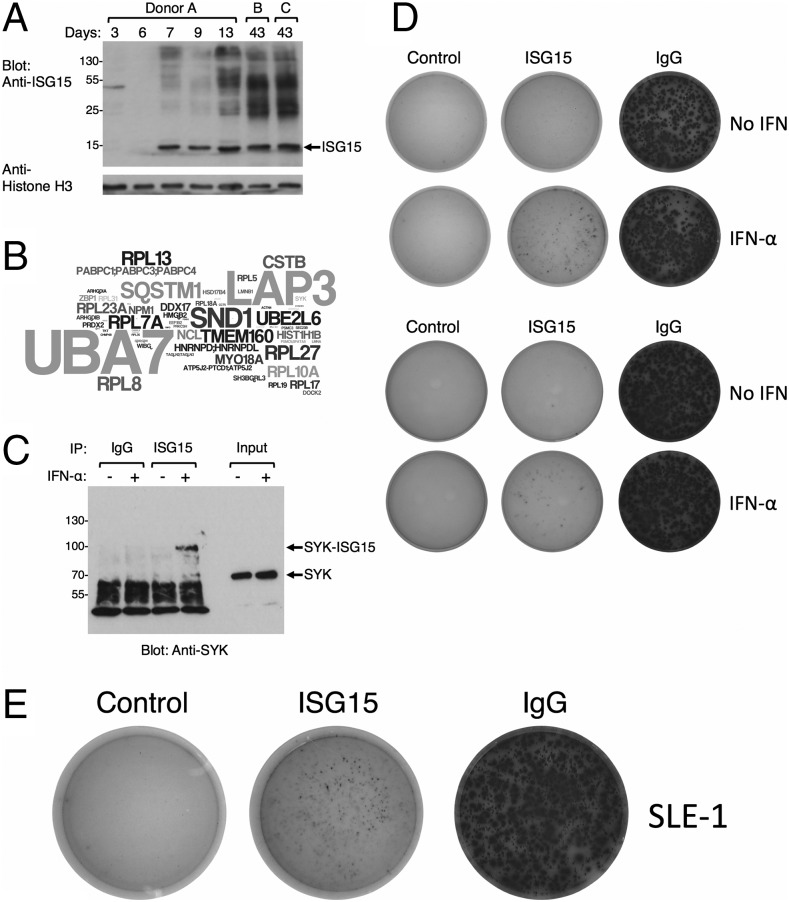
ASCs show evidence of protein ISGylation and ISG15 secretion. (**A**) ASCs generated in the presence of IFN-α show induction of unconjugated ISG15 and higher m.w. ISGylated proteins analyzed by Western blotting with anti-ISG15. (**B**) Mass spectrometry performed on samples immunoprecipitated with anti-ISG15 identifies substrates of ISGylation in plasmablasts stimulated with IFN-α for 24 h. Identified proteins are shown as a wordle with the number of peptide hits represented by font size. (**C**) Validation of ISGylation of SYK. Lysates generated from IFN-α–stimulated plasmablasts were immunoprecipitated (IP) with control rabbit IgG or anti-ISG15 and evaluated by Western blotting with anti-SYK. (**D**) Top panel, Secreted ISG15 detected by ELISPOT in plasmablast cultures derived from purified B cells in vitro, 20 h after exposure to IFN-α. Detection of secreted IgG in replicate cultures is shown for comparison. (D) Bottom panel, Plasmablasts purified from the peripheral blood of healthy volunteers were assayed for ISG15 production by ELISPOT 24 h after incubation in media alone or containing IFN-α. (**E**) Representative ELISPOT detecting spontaneous production of ISG15 by plasmablasts obtained from the peripheral blood of a patient with SLE.

ISG15 is highly induced in a number of cell types in response to type I IFNs, where it can act as an ubiquitin-like molecule that covalently attaches to Lys residues in a diverse range of target proteins in a process termed ISGylation (16). In mice, ISG15 has been shown to have a protective role in response to a variety of virus infections, although the precise mechanism remains unknown (56). To determine whether ASCs exposed to IFN-α showed evidence of a functional ISG15-conjugation pathway, we performed Western blotting on lysates generated from in vitro–differentiated cells. Prior to IFN-α treatment, there was no detectable ISG15 (Fig. 7A). However, consistent with the gene expression data by 24 h after IFN-α treatment, a prominent band corresponding to free intracellular ISG15 was observed. Additionally, we detected several high m.w. species, consistent with ISGylated protein conjugates. This pattern was preserved throughout the time course that paralleled our gene expression studies. We also evaluated the pattern in more differentiated PCs obtained after 40 d of culture in the presence of IFN-α. These cells display a phenotype resembling bone marrow PC with high levels of CD138 (data not shown). Notably, there are a wide range of ISGylated proteins, with pronounced species in the molecular mass range of 25–50 kDa, indicating that the process of ISGylation is maintained in PCs with sustained exposure to IFN-α.

A number of viral proteins have been shown to be ISGylated, and two proteomic studies have characterized substrates from IFN-treated cells (57, 58). To determine the nature of ISGylated proteins in the primary human plasmablasts generated in our system, we performed mass spectrometric analysis on anti-ISG15 immunoprecipitates. Consistent with previous publications, we detected numerous ribosomal proteins and components of the ISG15 pathway, including UBA7, the ISG15-activating enzyme, and UBE2L6, the ISG15-conjugating enzyme (Fig. 7B, Supplemental Table III). Among the targets, we also identified peptides from proteins of particular relevance to the B cell lineage, including SYK, IRF4, and DOCK2. To confirm that ISGylation may be involved in lineage-specific regulation, we evaluated SYK for evidence of modification in IFN-α–stimulated plasmablasts. Immunoprecipitation with anti-ISG15 followed by blotting with anti-SYK revealed a band with a molecular mass of ∼95 kDa, consistent with the conjugation of ISG15 to endogenous SYK (Fig. 7C). Thus, plasmablasts generated in the presence of IFN-α express both free ISG15 and a range of proteins covalently modified by the addition of ISG15 with the potential for lineage-specific effects.

### ISG15 secretion characterizes inflammatory plasmablasts

In addition to its role in the modification of intracellular substrates, ISG15 has been detected as a soluble product, despite the lack of apparent signal peptide to direct secretion (59, 60). The primary effect of extracellular free ISG15 defined to date is the stimulation of IFN-γ production (17, 18). In a recent paper, Bogunovic et al. (19) identified *ISG15* deficiency as the underlying genetic defect associated with enhanced susceptibility to mycobacterial disease. In this report, the lack of secretion of ISG15, principally attributed to granulocytes, was shown to lead to a failure of NK cell–derived IFN-γ production and loss of control of mycobacterial infection. The sustained and prominent induction of ISG15 among PCs cultured in the presence of IFN-α is therefore of particular relevance and if secreted in vivo would potentially place such PCs alongside granulocytes as a bridge between type 1 and type 2 IFN responses. We therefore assessed whether plasmablasts were capable of secreting ISG15.

To directly monitor secretion of ISG15, we developed an ELISPOT assay to interrogate secretion from in vitro–generated plasmablasts, which were cultured in the presence or absence of IFN-α for 20 h on anti-ISG15 capture wells. Cells grown in the absence of IFN-α produced copious amounts of IgG, but did not secrete appreciable amounts of ISG15. In contrast, plasmablasts grown in media containing IFN-α demonstrated active secretion of ISG15, readily detectable by ELISPOT (Fig. 7D, top panel). We then measured the production of soluble ISG15 from plasmablasts isolated from the peripheral blood of healthy volunteers. In one instance, we were able to detect ISG15 secretion from unmanipulated plasmablasts; however, ex vivo stimulation with IFN-α resulted in increased secretion (Fig. 7D, bottom panel). The variability of ISG15 secretion from freshly isolated plasmablasts suggests that prior in vivo exposure to IFN-α can be associated with polarization for ISG15 secretion.

The association between IFN-α and plasmablast production of ISG15 has the potential to implicate plasmablasts/PCs in a range of inflammatory processes. In terms of specific disease processes, SLE provides a prime example in which an association between autoantibody-secreting PCs and IFN-α–driven immune pathology are linked. SLE is characterized by a diverse range of autoantibodies, with recognition of at least 180 different Ags described in patients with SLE (61). Recent clinical success with biologic therapies such as rituximab (anti-CD20), belimumab (anti-BAFF), and epratuzamab (anti-CD22) also strongly implicate a B cell component in the disease (62). However, classic serological markers of disease (such as anti-dsDNA Abs and complement) and clinical status are frequently discordant (63). Regulation of B cell–targeted biologics only partially normalizes these biomarkers, which appears neither necessary nor sufficient for clinical response (64, 65). Hence, in addition to the pathogenic role of autoantibodies themselves, the potential involvement of the B cell lineage in disease biology in a fashion independent of Ag specificity would be of great interest.

A striking feature of the modular network analysis was the strong overlap of IFN-associated PC modules with signatures derived from the peripheral blood of patients with SLE as indicators of disease activity (30). Although IFN-α plays a central role, other studies in patients with SLE also point to an association with IFN-γ signaling. Given the potential interplay between IFN-α–mediated induction of soluble ISG15 and subsequent IFN-γ production by responding NK or T cells, we therefore directly evaluated whether plasmablasts present in the peripheral blood of patients with SLE showed evidence of ISG15 secretion. Plasmablasts from patients with active disease were identified and sorted based on surface phenotype of CD19^+^CD38^hi^CD27^hi^ and assayed by ELISPOT (Fig. 7E). ISG15 secretion was detected in 8 out of 10 samples and was particularly notable in a patient with very active disease (Supplemental Table IV). These data confirm that SLE is characterized by the generation of a proinflammatory ASC population that secretes ISG15 and identify a novel mechanism by which inflammatory ISG15-secreting ASCs may contribute to pathogenesis in a fashion that is independent of Ig.

## Discussion

Protective immunity in part hinges on the successful and appropriate production of Abs. Particular biological contexts can elicit highly variable Ab responses in terms of magnitude, kinetics, affinity, and isotype produced. In each scenario, the outcome is dependent upon the ordered process of transforming an activated B cell into an Ab-secreting PC. During this transition, transcriptional programs governing the B cell phenotype give way to the program that defines secretory potential. The extent to which PCs may acquire the capacity to secrete cytokines and other immune modulatory products during this differentiation process is only just beginning to be elucidated. Furthermore, the extent to which such non-Ig secretory capacity may be imposed upon differentiated ASCs rather than determined by the nature of the immune stimulus initiating differentiation has not been defined. Given the dedicated secretory capacity of the mature PC and the potential of such cells for extended survival, such function would provide a significant contribution to modulation of inflammatory and immune responses. The analysis presented in this study points to the capacity of human PCs to be polarized for proinflammatory immune modulatory function in response to their cytokine environment in disease. This polarization for proinflammatory function is superimposed as part of a distinct gene expression module onto the programs governing the primary facets of the PC state. This establishes a paradigm for functional polarization of differentiated PCs in response to their microenvironments and points to the potential existence of a range of functionally distinct ASCs.

Many of the central players essential for the differentiation of B cells to PCs have been determined. However, less is known about the impact of individual biological contexts on the underlying regulatory programs. A recent study from Corcoran and colleagues has provided tantalizing insights into the nature of wider transcriptional differences obtained in different ASC populations (66). For example, the authors identified that the tissue residence of the evaluated PC biased the underlying gene expression patterns. Additionally, they found that ASCs generated in vitro by exposure of murine B cells to either the T-I stimulus LPS or the T-D mimic CD40L/IL-4/IL-5 displayed a less mature transcriptional profile when compared with in vivo counterparts, but were also characterized by ∼200 unique stimulus-dependent transcripts. Our data now provide direct evidence that ASC populations may be modified by inflammatory signals to induce functional gene modules superimposed on the core ASC state. Rather than examine a purified population of phenotypically mature PCs that have reached some degree of transcriptional stasis, we elected to perform a detailed temporal analysis of all responding cells to capture the dynamics of the response. The data sets were subjected to weighted gene correlation network analysis to define groups of coordinately regulated genes and highlight pathways operating during differentiation. This analysis has the potential to uncover previously unanticipated players in the regulation of PC differentiation, thus providing a resource for further exploration of potential core transcriptional regulators of PC differentiation.

Moreover, rather than comparing the effect of initial stimuli that impose a change on B cells, we instead focused on the window of plasmablast to PC transition. We reasoned that plasmablasts are likely to encounter novel signals as they transit from the site of initial stimulation that may have short- or long-term effects on gene expression. In particular, we were interested in the effect of IFN, given its association with PC survival and autoimmune pathology. The emergence of the ISG15-conjugating system as the most prominent pathway associated with IFN-α–treated plasmablasts suggested that ISG15 or modification of cellular proteins by ISG15 could potentially alter functional output. Upon examination of IFN-α–stimulated plasmablasts, we detected a rapid and prolonged induction of both unconjugated ISG15 and numerous ISGylated substrates. In PCs that were maintained in the presence of IFN-α for an extended period (36 d), prominent ISGylated proteins were detected, indicating that sustained contact with IFN-α leads to a gradual increase in the accumulation of ISGylated proteins. Previous studies investigating the effect of modification of proteins by ISG15 have identified a range of effects. For example, ISGylation of 4EHP enhances m^7^GTP cap structure-binding activity to suppress translation (67), whereas modification of IRF3 inhibits ubiquitin-mediated degradation resulting in sustained activation (68). In most instances, the focus has been on the impact of ISG15 on the antiviral properties imparted by IFN-α. To explore the consequences of expressing ISG15 in a setting detached from infection, we performed mass spectrometry on ISG15 immunoprecipates prepared from IFN-α treated plasmablasts. Although the majority of targets were consistent with previous reports, we identified several novel ISGylated proteins of particular relevance to the B-cell lineage and confirmed that a fraction of the tyrosine kinase SYK contains this previously uncharacterized posttranslational modification. The impact on SYK function remains to be determined; however, ISG15 is conjugated to lysine residues of target substrate proteins in a similar fashion to ubiquitin. As SYK has previously been shown to be ubiquitinated after GPVI activation in platelets resulting in enhanced activity (69), it is tempting to speculate that ISG15 modification has an analogous effect. Indeed, a unique population of memory-like B cells expressing high levels of SYK has been identified in patients with SLE (70), in whom exposure to IFN-α drives expression of ISG15.

Most of the work to date on ISG15 has focused on the intracellular modification of proteins; however, recent data have revitalized an interest in the function of the secreted molecule. The identification of patients lacking ISG15 has confirmed in vitro observations that production of IFN-γ is a major physiological consequence of encounter with secreted ISG15 (19). In the setting of mycobacterial infection, lack of ISG15 secretion in affected patients was largely attributed to production by neutrophils, although lymphocyte populations also have the capacity to secrete ISG15. Our data now document the capacity of plasmablasts and PCs, cell types specialized for high-level secretion, to produce soluble ISG15. Most importantly, we provide evidence of ISG15 secretion from ASCs in patients with active SLE, thus linking this aspect of ISG15 function to inflammatory disease.

Murine models of lupus and genetic association studies have long indicated a role for type 1 IFN in the pathogenesis of SLE (10), and endeavors characterizing type 1 IFN–induced transcripts in blood and tissue samples from patients with SLE have confirmed a significant connection to IFN-α in particular (38–41). Although IFN-α has been considered to lie at the heart of lupus-associated pathology, there have been a number of reports that provide evidence for a contribution to the disease-associated signature by type 2 IFN (71–73). Most recently, Chichel et al. (30) have employed modular transcriptional repertoire analysis to monitor the dynamics of IFN signatures across patients with SLE and within individuals over time. They identified three distinct modules that were differentially associated with disease severity, with notable representation of IFN-β– and IFN-γ–driven transcripts in the modules typifying patients with active disease. Thus, the progression of the disease may mirror a transition from type 1 to type 2 IFN involvement. Given that, the SLE IFN signature also contains a PC-related module absent in other diseases with prominent IFN-inducible gene profiles such as tuberculosis (29). Indeed, it has been shown that CD19-enriched cells are a source of IFN module gene expression in SLE, including ISG15 and correlating with the SLE Disease Activity Index score (74). Moreover, in active SLE, CD19-enriched cells also display gene expression patterns consistent with ASC differentiation. Taken in conjunction with our data, this would be consistent with the principle origin of IFN signatures from CD19^+^ ASCs. Because ISG15 can act as a bridge between type 1 and type 2 IFN through the activation of NK cells and T cells, these data fit with a model in which the secretion of soluble ISG15 by ASCs contributes to the shift toward IFN-γ in more aggressive disease.

The secretion of functional products other than Ig by PCs has now been documented in range of disease contexts, including the development of an SLE-like disease in *Lyn*-deficient mice. In this instance, the secretion of IL-10 by ASCs provided protection against inflammation caused by myeloid and T cell activation (75). Immunosuppressive PCs have also been demonstrated in a murine model of *Salmonella* Typhimurium infection in which IL-10–expressing CD19^+^CD138^+^ cells were detected within 24 h of administering the bacteria (8). This finding was solidified in a further study that matched the level of IL-10 secretion to the maturation stage of ASC populations in animals challenged with *Salmonella* (5). The phenotypically most mature cells that expressed high levels of *Prdm1* and *Irf4* also expressed the highest levels of IL-10. These results were bolstered by a similar observation in the setting of experimental autoimmune encephalomyelitis in which CD138^hi^ PCs were the major B cell–derived source of IL-10. Moreover, PCs in both models of disease also produced the immunosuppressive cytokine IL-35; however, the two cytokines were not detected simultaneously, suggesting that PCs or their precursors might differentially respond to initial triggers.

The above models suggest a strong association between PCs and the suppression of inflammation. However, cytokine production by PCs is not limited to those with anti-inflammatory properties. In two further murine models that generate polyclonal PC responses, there is strong evidence that the major source of critical proinflammatory cytokines is a PC population. In the first instance, the authors described the contribution of PC-produced IL-17 to the control of acute *Trypanonsoma cruzi* infection (4). In this report, a *trans*-sialidase produced by the parasite was identified that could directly stimulate primary mouse or human B cells to make IL-17 using a retinoic acid–related orphan receptor γt–independent transcriptional program. In the second example, IgM-secreting CD138^+^ PCs were shown to account for ∼75% of GM-CSF–expressing cells in the spleen in a murine model of sepsis (76). Deletion of these cells had a profound impact on bacterial clearance and culminated in septic shock.

Not only do PCs contribute in novel ways during infection, they also participate in maintaining the homeostatic balance of the gastrointestinal tract. The production of IgA by mucosal-associated PCs is an essential component of gut homeostasis and is initiated by exposure of B cells to inducible NO synthase and TNF-α. Surprisingly, IgA^+^ PCs resident in the lamina propria of the small intestine were shown to be the primary local source for both these pathways (3). An engineered inability of B-lineage cells to secrete inducible NO synthase and TNF-α had a compound effect on gut biology, including loss of IgA production, altered microbiota composition, and diminished response to the pathogen *Citrobacter rodentium*. Thus, PCs in the microenvironment of the gut are identified as crucial effectors, both in terms of the Ig as well as the cytokines they secrete.

The current body of data indicate a diverse role for ASCs beyond the primary task of Ig production. Although most of the examples described thus far arise in the context of stimulation through microbial-derived molecules, it seems likely that alternative signals will generate PCs with distinct secretory profiles. In particular, our data are consistent with an inflammatory niche-driven generation of ISG15-secreting PCs. Additionally, the documentation of activating/prosurvival signals provided by diverse cells contributing to the multicomponent PC niche (77), such as eosinophils, megakaryocytes, and basophils, suggests that other settings may be able to impart unique functional capabilities to the responding PCs (78, 79). Given the new range of activities that can be attributed to ASCs, one of the challenges will be to determine the extent to which these cells remain committed to a particular phenotype. One can envision at least three scenarios that give rise to functionally distinct PC subsets: 1) a deterministic model in which B cells with a pre-established program differentiate into a fixed polarized effector; 2) an instructive model in which B cells initiate differentiation in a polarizing context leading to a fixed polarized effector; or 3) a responsive model in which established ASCs are exposed to a polarizing context that generates labile polarized effectors. Our results suggest that cells committed to Ab secretion are indeed malleable in different environmental conditions and point to as yet undiscovered roles for PC contribution to immune regulation.

## Supplementary Material

Data Supplement
