# Supplementary material for: Network Analysis Identifies Proinflammatory Plasma Cell Polarization for Secretion of ISG15 in Human Autoimmunity
Source: J Immunol. 2016 Jun 29;197(4):1447–59. doi: 10.4049/jimmunol.1600624 (PMC4974491; doi:10.4049/jimmunol.1600624)
Supplement: Data Supplement [file JI_1600624.zip › JI_1600624_Supplemental_Material.pdf]

Supplemental Table 1:

Lists the genes differentially expressed at each of the sampled time points.

Supplemental Table 2:

Lists the genes associated with each module and individual connectivity values.

Supplemental Table 3:

Lists the ISGylated proteins identified by mass spectrometry.

Supplemental Table 4:

Clinical characteristics of SLE patients and plasmablast ISG15 secretion.
