# Supplementary material for: Network Analysis Identifies Proinflammatory Plasma Cell Polarization for Secretion of ISG15 in Human Autoimmunity
Source: J Immunol. 2016 Jun 29;197(4):1447–59. doi: 10.4049/jimmunol.1600624 (PMC4974491; doi:10.4049/jimmunol.1600624)
Supplement: Data Supplement [file JI_1600624.zip › JI_1600624_Supplemental_Table_4.pdf]

Supplemental Table 4.
Clinical characteristics of SLE patients and ISG15 secretion

Sample ID	ACR/ SLICC Criteria	Age	Gender	dsDNA	RiboP	Ro60	RNP	Ro52	Sm	Jo1	La	Sm/RNP	Chromatin	C3	C4	IgM	IgA	IgG	SLEDAI Score	BILAG Score	ELISpot Score*
**SLE-1**	Y	51	F	301	0.2	9	0.4	1.6	9	0.2	1.5	9	9	0.56	0.12	0.49	4.38	23.1	16	25	8.1
**SLE-2**	Y	38	F	0	0	0	0	9	0.2	0	0	9	6	1.48	0.31	0.84	3.17	32.1	4	9	5
**SLE-3**	N	65	F	0	0	0	0	0	0	0	0	1.9	0	1.48	0.3	0.54	2.29	14.5	4	1	3.6
**SLE-4**	Y	52	F	0	0	9	0	0	0	0	0	0	0	1.18	0.16	0.51	1.98	6.5	4	1	1.7
**SLE-5**	Y		F	301	0.3	9	0	9	0.4	0	9	0	9	0.5	0	1.12	5.16	31.1	10	13	1.9
**SLE-6**	N	44	F	32	0	0	0	0	0	0	0	0	0	1.33	0.28	1.08	1.72	13.5	6	2	1.7
**SLE-7**	Y	32	F	73	0	0	0	0	0	0	0	0	0	1.15	0.27	0.89	2.59	9.3	8	3	0.7
**SLE-8**	Y	48	F	124	0	0	0	0	0	0	0	0	1.2	0.77	0.09	0.07	1.78	6.1	10	8	7.9
**SLE-9**	Y	44	F	0	0	0	0	9	0	0	0	0	0	1.26	0.28	1.04	1.66	11	6	9	2.7
**SLE-10**	Y	42	F	0	0	0	0	0	0	0	0	0	2	0.69	0.13	2.45	2.87	13.6	2	1	1

*The ELISpot score was derived from tabulating the number of spots in a well compared to the number present in a control sample (GM12878 cells). This ratio was then normalized for the number of plasmablasts that were seeded into the ELISpot.
